# RESPONSIVENESS OF UPPER LIMB SCALES AND TRUNK CONTROL FOR THE EVOLUTION OF PATIENTS WITH DUCHENNE MUSCULAR DYSTROPHY

**DOI:** 10.1590/1984-0462/2021/39/2020045

**Published:** 2021-02-24

**Authors:** Flaviana Kelly de Lima Maciel, Ana Lúcia Yaeko da Silva Santos, Cristina dos Santos Cardoso de Sá

**Affiliations:** a Universidade Federal de São Paulo, São Paulo, SP, Brazil.; b Universidade Anhanguera, São Paulo, SP, Brazil.

**Keywords:** Muscular dystrophy, Duchenne, Clinical evolution, Upper extremity, Postural balance, Posture, Distrofia muscular de Duchenne, Evolução clínica, Extremidade superior, Equilíbrio postural, Postura

## Abstract

**Objective:**

To verify the interval of responsiveness to the scales Segmental Assessment of Trunk Control (SATCo-BR), Performance of Upper Limbs (PUL), and Jebsen Taylor Test (JTT) in patients with Duchenne Muscular Dystrophy (DMD).

**Methods:**

We assessed patients with DMD aged 6 to 19 years old and with mini-mental (MMSE) score above 10 points. The assessments were performed individually, in a single session. The upper limb function was performed by PUL and JTT, and trunk control by SATCo-BR. Assessments were repeated six and 12 months after the initial assessment. The repeated-measures analysis of variance model and Bonferroni’s multiple comparison method were employed as *post hoc* analysis; when the ANOVA assumptions were not met, the Friedman test was applied.

**Results:**

The sample consisted of 28 patients evaluated in three moments (initial, and six and 12 months after the beginning). There was a time effect for the Upper Limb function performance in the total JTT, and for the subtests, except for subtests 1 and 6, which did not show a difference between the different moments. There was also a time effect for the score of total PUL, proximal PUL, intermediate PUL, and distal PUL. In the SATCo-BR, this effect was observed between the initial and 6 months, and between the initial and 12 months.

**Conclusions:**

The JTT, PUL, and SATCo-BR scales can detect changes over time, and they showed responsiveness to detect the evolution of the disease in the 6-month interval.

## RESUMO

**Objetivo:**

Verificar o intervalo de tempo para a responsividade das escalas Segmental Assessment of Trunk Control (SATCo-BR), Performance of Upper Limb (PUL) e o Teste de Função Manual de Jebsen Taylor (TJT) em pacientes com distrofia muscular de Duchenne (DMD).

**Métodos:**

Foram avaliados pacientes com DMD nas idades entre 6 e 19 anos, e com escore do Mini Exame do Estado Mental (MEEM) a partir de 10 pontos. As avaliações foram realizadas individualmente, em uma única sessão: a função de membro superior (MS) ocorreu pela PUL e TJT; e da do controle de tronco, pela SATCo-BR. As avaliações foram repetidas após seis e 12 meses da avaliação inicial. Foi empregado o modelo de análise de variância com medidas repetidas e o método de comparações múltiplas de Bonferroni, como análise post hoc; quando os pressupostos da ANOVA não foram atendidos, foi aplicado o teste de Friedman.

**Resultados:**

A amostra foi composta por 28 pacientes avaliados em três momentos (inicial, após seis meses e após 12 meses). Houve efeito do tempo no desempenho da função Membro Superior no TJT total e nos subtestes, exceto nos subtestes 1 e 6, que não apresentaram diferença nas avaliações entre os diferentes momentos. Houve efeito do tempo para o escore da PUL total, PUL proximal, PUL intermediário e PUL distal. No SATCo-BR, esse efeito foi entre o inicial e após seis meses, e entre o inicial e após 12 meses.

**Conclusões:**

As escalas TJT, PUL e SATCo-BR são capazes de detectar alterações ao longo do tempo, e apresentam responsividade para detectar a evolução da doença em intervalo de 6 meses.

## INTRODUCTION

Duchenne muscular dystrophy (DMD) is the most common muscular dystrophy, affecting 1/3600 live births.^1^ The progressive and irreversible character of the clinical manifestations of DMD begins around the first three years of life, with the symmetrical and bilateral weakening of the musculature. This muscle weakness is evident, as the child finds it difficult to run, get off the floor, go up and down the stairs, and frequent falls,^1^ ^,^ ^2^ progressing to trunk, and upper (UL) and lower limb (LL) musculature.^1^ ^,^ ^2^ ^,^ ^3^ ^,^ ^4^

The decrease in muscle strength^5^ increases difficulties in daily, social, and professional life, impairing the functional independence and the quality of life of these patients.^6^ Therefore, the use of UL for patients with DMD is indispensable for the long-awaited functional independence since the UL function establishes the relationship of the individual with the environment.^7^ Besides UL ability, performance in activities of daily living depends on postural adjustments, which becomes deficient in individuals with DMD due to trunk muscle imbalance as muscle weakness progresses.^3^

There has been a constant search for methods that can quantify and monitor the evolution of muscle strength and motor function of these patients.^8^ Some functional assessment scales have been validated for DMD, such as the Vignos Scale, which is a rating system;^9^ the Jebsen-Taylor Test (JTT) that assesses the UL function concerning the execution time of tasks,^7^ ^,^ ^10^ and Performance of Upper Limb (PUL) test, which evaluates the UL function, in terms of movement quality.^11^ ^,^ ^12^ The Trunk Control Segmental Assessment (SATCo) assesses the level of trunk control.^3^ ^,^ ^13^

Therefore, for an assessment instrument to be recognized for its quality, it must be evaluated for psychometric properties.^14^ When compared to the study of validity and reliability, responsiveness analysis is less studied, despite its importance.^15^ To be responsive, an instrument must correspond to an appropriate statistical measure, capable of detecting changes in the functional capacity of patients over time,^16^ which is of great relevance to the daily clinical practice of the physical therapist, as it allows the identification of small changes in the clinical condition of these patients, as well as their ability to respond after interventions. We aim to verify the time interval in which the scales to assess trunk control and upper limb function are responsive to detect progression in patients with DMD.

## METHOD

This is a longitudinal study, whose assessments were performed at the Muscular Dystrophy Clinic of the Neuromuscular Diseases Research Sector, that was approved by the Research Ethics Committee of the University (no. 0911/2017). The parents and/or guardians and those over 18 agreed to participate by signing the informed consent form, and, in the case of minors under the age of 18 years, the agreement was also obtained by the assent form.

The study comprised 28 patients diagnosed with muscle biopsy-confirmed DMD or genetic testing, aged 6 to 19 years (12.1±3.6), all male. The sample size, a priori, was composed of 28 volunteers. The sample size calculation was performed according to a score of the upper limb function used on the PUL scale in patients with DMD^10^ (power 0.80; significance level 0.05; effect size=0.025), through the G-Power version 3.1.1.

The study excluded patients who underwent previous surgical procedures in the ULs and/or spine, or with associated orthopedic and/or neurological diseases; with difficulty in understanding simple verbal commands, or volunteers with cognitive deficits, that is, with a Mini-Mental State Examination (MMSE) score lower than 10 points,^16^ visual and/or hearing deficits that made it impossible to apply the research protocol or those who, for any reason, interrupted the assessment.

The cognitive aspect was assessed by applying the MMSE, which has been validated for the Brazilian population,^17^ and was recommended by the Brazilian DMD Consensus to assess cognitive performance.^18^ In our study, we set a minimum score of 10 points, this is the minimum grade for DMD patients to understand simple verbal commands.^16^ The MMSE is a test designed to assess specific cognitive functions (temporal orientation, spatial orientation, memory, attention and calculation, three-word recall, language, and constructive visual capacity). The maximum MMSE score is 30 points, with zero indicating the highest degree of cognitive impairment and 30 points indicating the best cognitive ability.^17^

The Vignos Scale was used to classify or stage the disease of the DMD individuals. The scale score ranges from 0 to 10; the higher the score, the worse the individual’s functional performance.^9^ This score classified the participants in walking (Vignos≤6) or non-walking (Vignos≥7) groups.

The SATCo is a clinical analysis tool that assesses the trunk control, which was developed and validated by Butler et al.^13^ This scale was translated into Brazilian Portuguese.^19^ For this application, we used a height-adjustable seat, a strap system attached to the patient, and the seat that allowed the footrest to ensure that the pelvis was kept in a neutral position. With the patient sitting on the backless bench, with the LL propped on the floor and strapped in, the evaluator progressively reduces the support of his or her hands, from the full support position to the non-support position, characterized by the level of trunk control, which varies from head control to full trunk control, with level-1 corresponding to head control, level-2 to upper thoracic, level-3 to mid-thoracic, level-4 to lower thoracic, level-5 to upper lumbar, level-6 to lower lumbar, and level-7 to full trunk control. To define each individual’s trunk control level, the last level at which all three control components (static, active, and reactive) were present was considered.^13^ Sá et al.^3^ identified the need for adaptation concerning the positioning of the ULs for this population. From the upper thoracic control level, patients should maintain shoulder abduction of at least 45°.

The JTT assesses the manual function often used in activities of daily living and objectively measures performance in functional tasks. It is composed of seven subtests to assess most components of the manual function:^20^ written subtest (1JTT); flip card (2JTT); to take ordinary small objects and place them inside a pot (3JTT); simulates feeding (4JTT); to pick up checkers game pieces and stack them (5JTT); to pick up wide and light objects (6JTT); taking large and weighted objects (7JTT); this subtest was excluded due to the patients’ difficulty in performing activities with weight.^8^ ^,^ ^10^ To perform the JTT with the above adaptations, instructions on the specific mode of execution of each subtest were provided before its start. No prior attempt was allowed as a form of training. All subtests were performed in the sequence established for the dominant UL. When a volunteer failed to complete the attempt, it was excluded, and timing was not analyzed. Each attempt was quantitatively measured using a stopwatch. The subtests were filmed, and the time of all attempts was measured in seconds using a stopwatch.^20^ ^,^ ^21^. The test has normative data for healthy people.^22^

The PUL, version 1.2, was created to assess both walking and non-walking patients, based on the natural progression of DMD.^11^ Divided into proximal (shoulder), intermediate (elbow), and distal (wrist) levels, it comprises 22 items, the first of which defines the patient’s functional level, that is, if in this item, the patient gets a score lower than 4 points, the evaluator starts the assessment by the average level. The score in each level varies with the task, from 0 to 1 until 0 to 5.^12^ The maximum score of the scale comprises 74 points (best UL performance).^12^ Non-walking DMD individuals were evaluated seated in their wheelchairs, and the walking ones, seated in a chair with a backrest. All subjects were positioned at a 90º hip, knee, and ankle flexion. For better data evaluation, a camera was positioned 2 meters from the subject, and the footage was analyzed for scoring.

At the proximal level, the flexion and abduction of the shoulder without and with weight (200, 500, or 1000g) were assessed. At the intermediate level, the ability to bend and extend the elbow without and with weight for functional tasks, carry a weighted cup (50 or 200g) to the mouth, put both hands simultaneously on the table, lift a previous weight (100, 200, 500, or 1000g), lift light and heavy cans in multiple directions, stack light, and heavy cans, open a pot and tear a paper folded in 4. At the distal level, the forearm skills, pronation, and supination are addressed; wrist: flexion, extension, radial, and ulnar deviation; and fingers: flexion, extension, adduction, abduction, combined by manipulation, bi-digital, and tri-digital tweezer grasp. All tasks were performed with the dominant UL except for bimanual tasks.

When performing the MMSE and Vignos, each participant performed the following assessments: SATCo-BR, JTT, and PUL, and returned to repeat them after six and 12 months from the initial assessment. The period of six and 12 months after the initial assessment was defined based on the study by Carvalho et al.^23^ which revealed low to moderate responsiveness for the assessment of gait with reevaluations performed after three months. In the period between evaluations, most patients maintained their routine of interventions, two 45-minute physical therapy sessions per week. To perform these assessments, patients were seated, and we used a camera, Sony Hdr-cx405 Full HD Digital Zoom 350x+32gb camcorder, mounted on a Canon Nikon Sony professional photographic universal tripod, and installed at 2 m from the dominant UL side of the patients to film the assessments. After all assessments, the videos of each patient were analyzed by the same examiner, which allowed identifying the patient’s trunk control level; the performance of the UL function by counting the execution time of each task in seconds (JTT subtest), using the timer of an iPhone 6S; and the quality of the movements by PUL.

The Shapiro-Wilk test was applied to test data normality, the Levene test to assess the homogeneity of variables, and the Mauchly test to assess the sphericity. To compare the assessments of the variables, total JTT, and other JTT subtests, the repeated-measures analysis of variance model (ANOVA) and Bonferroni’s multiple comparisons method were used as *post hoc* analysis. For the variable, trunk control level, the Friedman test was applied. Data were analyzed using the R i386 software, version 3.5.2. Values p≤0.05 were considered significant.

## RESULTS

Table 1 shows the characterization of the 28 patients during all assessments in relation to age, weight, height, body mass index (BMI), and MMSE score (Table 1).

**Table 1:** Patient characterization: age, anthropometric measurements according to the assessments (initial, after six months, and after 12 months), and Mini-Mental State Examination.

	Assessment 1 (initial)	Assessment 2 (after 6 months)	Assessment 3 (after 12 months)
	Mean±SD	Mean±SD	Mean±SD
Age (years)	12.1±3.5	12.4±3.4	12.6±3.5
Weight (kg)	47.8±15.7	48.5±16.1	50.7±16.8
Height (m)	1.50±0.2	1.51±0.2	1.54±0.2
BMI	20.7±4.7	20.8±5.4	21.0±5.7
MMSE	23.8±5.1	-	-

SD: standard deviation; BMI: body mass index; MMSE: Mini-Mental State Examination.

Regarding the disease staging, it was noted, through the Vignos scale classification, that 46.4% of the patients in the initial assessment had Vignos 7 (they are in a wheelchair, sit upright, can touch the chair, and can perform activities of daily living in the chair or bed); from the second (50%) and third assessments, this percentage increased to over 53.5% (Table 2). Among patients using medication (89.3%), 23 were receiving steroids, three were receiving Ataluren, 12 were receiving antihypertensive, and 8 were receiving antiosteoporosis.

**Table 2:** Patient characterization: treatment and Vignos Scale according to the assessments (initial, after six months, and after 12 months).

	Assessment 1 (initial)				Assessment 2 (after 6 months)				Assessment 3 (after 12 months)			
	Yes		No		Yes		No		Yes		No	
	n	(%)	n	(%)	n	(%)	n	(%)	n	%)	n	(%)
Physical therapy	23	(82.1)	5	(17.9)	21	(75.0)	7	(25.0)	21	(75.0)	7	(25.0)
Orthosis	11	(39.3)	17	(60.7)	13	(46.4)	15	(53.6)	12	(42.9)	16	(68.1)
Scoliosis	15	(53.8)	13	(46.2)								
Drugs	25	(89.3)	3	(10.7)								
Vignos scale					0	(0)			0	(0)		
1	0	(0)			1	3.5			1	(3.5)		
2	2	(7.1)										
3	2	(7.1)			2	7.1			1	(3.5)		
4	5	(17.9)			6	21.4			4	(14.3)		
5	0	(0)			0	(0)			1	(3.5)		
6	2	(7.1)			0	(0)			1	(3.5)		
7	13	(46.4)			14	(50.0)			15	(53.5)		
8	4	(14.3)			4	(12.3)			3	(10.7)		
9	0	(0)			1	(3.5)			2	(7.19)		

n: number of individuals.

There was an effect of the time on the performance of the ULs in total JTT (F_2,54_=3.442; p=0.001), indicating the JTT instrument can detect changes in the evolution of DMD patients over time, and for the following JTT subtests: 2JTT(F_2.54_=4.182; p=0.02), 3JTT (F_2.54_=8.385; p=0.003), 4JTT (F_2.54_=3.235; p=0.047), and 5JTT (F_2.54_=3.512; p=0.037). For 1JTT (F_2.54_=0.230; p=0.732) and 6JTT (F_2.54_=3.313; p=0.074) there was no significant difference in the assessments at different times (Table 3). Subsequently, Bonferroni’s multiple comparison analysis indicated that, for 2JTT, the difference occurred between the initial assessments and after 6 months (p=0.040) and between the initial assessments and after 12 months (p=0.010). For 3JTT, the difference was observed between the initial assessment and after six months (p=0.003) and between the initial assessments and after 12 months (p=0.001). For 4JTT, the difference occurred between the initial assessment and after 12 months (p=0.028), and for 5JTT, between the initial assessment and after six months (p=0.034), and between the initial assessment and after 12 months (p=0.021). For the total performance of the UL function by the JTT, the difference occurred between the initial assessment and after 12 months (p=0.009) (Table 3). Therefore, the 2JTT, 3JTT, and 5JTT allow us to identify the performance deterioration in the six and 12-month intervals from the initial assessment and 4JTT after 12 months from the initial assessment.

**Table 3:** Values presented as mean, standard deviation, median, and interquartile range according to the variables studied at different assessment times (analysis of variance, Friedman, and Bonferroni’s *post hoc* test, p≤0.05).

	Assessment 1 (initial)	Assessment 2 (after 6 months)	Assessment 3 (after 12 months)	p-value		
	Mean±SD	Mean±SD	Mean±SD	*	**	***
JTT Scale						
1JTT	63.8±43.8	62.7±40.2	64.7±40.9	ns	ns	ns
2JTT	13.6±5.3	15.8±9.5	16.2±7.0	0.04	0.01	0.708
3JTT	14.8±8.4	16.4± 9.5	18.1± 11.2	0.003	0.001	0.086
4JTT	27.3±14.4	28.4±12.9	33.7±20.7	0.655	0.028	0.075
5JTT	12.5±5.2	15.4±8.5	15.1±8.5	0.034	0.021	0.86
6JTT	12.5±9.6	13.8±9.9	17.1±18.5	ns	ns	ns
Total JTT	203.6 ±91.8	211.0±102.6	234.6±122.6	0.397	0.009	0.017
PUL Scale						
Proximal	4.0±4.1	2.9±3.3	2.2±3.1	0.005	0.001	0.003
Intermediate	15.1±8.2	12.3±6.6	10.1 ±5.4	0.001	0.001	0.001
Distal	22.5±1.7	21.5±1.9	20.5±1.8	0.001	0.001	0.001
Total	41.6±13.3	36.7±11.3	32.8±9.8	0.001	0.001	0.001

Assessment 1 (initial assessment) n=28; Assessment 2 (after six months) n=28; Assessment 3 (after 12 months) n=28; SD: standard deviation. Total JJT: Jebsen Taylor test; 1JTT: writing; 2JTT: flip cards; 3JTT: pick up small and common objects; 4JTT: simulate feeding; 5JTT: pick up checkers pieces; 6JTT: pick up large and light objects; PUL: Performance of Upper Limb; ns: Non-significant; *p-value between the initial assessment and after six months; **p-value between the initial assessment and after 12 months; ***p-value between six months and 12 months assessment.

When comparing the results of SATCo-BR, the Friedman test shows that there was an effect of the time (p=0.002) and that this effect occurs between the initial assessment and after six months (p=0.050), and between the initial assessment and after 12 months (p=0.050). Regarding the quality of the UL function observed by the PUL scale, there was also an effect of the time. This effect was observed in the total PUL score (p=0.001), as well as proximal PUL (p=0.001), intermediate PUL (p=0.001), and distal PUL (p=0.001). Comparisons between assessments that occurred at the three moments revealed the effect of time in the 6-month interval (Table 3).

## DISCUSSION

This study verified the time interval in which the scales for assessing trunk control and upper limb function are responsive to detect progression in patients with DMD. The results indicated changes in the function of the ULs during the proposed tasks over time and may be used at intervals of six months, allowing to detect some changes in this period by the measured quantitative JTT and the measured PUL, qualitative performance of UL, that is, there was responsiveness.

We observed that JTT did not identify a change between the 6-month assessments and the 12-month assessment. This possibly occurred, as the JTT measures the total execution time of the tasks, as well as the time of execution in each subtest. Therefore, JTT is a measure of performance. The non-change in the execution time of each task in the interval between six and 12 months, is not indicating that the disease does not evolve, but that the patients may be using compensatory strategies to perform the task.^20^ We can infer that the PUL scale, which measures the quality of the movement performed in each task, detected changes in the three assessments (initial, after six months and after 12 months).^24^

Thus, it is important to associate the two scales, JTT and PUL to identify changes in the evolution of upper limb function in patients with DMD. The JTT is an easy and quick assessment tool to administer and uses readily available materials,^20^ and the PUL scale allows one to identify the worsening of movement quality. PUL, in its version labeled 1.2, has proven to be reliable, reproducible, and suitable for international multicenter settings, designed to assess UL function in DMD, and thus be able to identify potential trajectories of disease progression.^24^

Besides monitoring aspects such as mobility and muscle strength, the ability of the ULs in the JTT can be influenced, among some components, by age, disease staging, and mental state.^20^ The 1JTT and 6JTT were the only ones that did not show differences in the assessments at different moments, as well as studies that correlated JTT with PUL. They compared walking and non-walking patients, finding no difference in the JTT between the groups assessed, assuming greater cognitive demand and motor coordination in this subtest.^6^ ^,^ ^7^ A study using the JTT showed that the test involves the recruitment and control of the proximal, medium, and distal levels.^25^ Thus, compensatory movements were observed because muscle weakness impairs UL performance and restricts movements, which stimulates the selection of new motor strategies.^26^ ^,^ ^27^ Functional motor loss in DMD usually occurs from proximal to distal, and a lack of proximal stability may impair the timed performance of the ULs.^27^ Muscle fatigue is another clinical aspect that can significantly interfere with the test application since this fatigue may increase after the muscle effort required to perform a task.^28^

The key tool used to assess trunk control in DMD patients,^3^ this study showed that SATCO is also an essential tool to detect changes in the level of functional trunk control of DMD patients throughout time; that the greatest impairment of trunk control is related to the phases after gait loss. We observed that, even with the progression of the disease and higher Vignos scores, at some point, when the disease progresses more slowly, these patients tend to stabilize the body in a sitting position and can perform more complex daily tasks.

Trunk control is a basic motor skill that depends on the integration of neural and musculoskeletal function and is crucial to perform a range of daily activities.^13^ The muscle weakness of this population destabilizes posture and may interfere with the movements of the ULs,^5^ ^,^ ^24^ which makes these patients dependent on their caregivers and interferes with their quality of life. SATCo-Br allows identifying the area of the trunk that has reduced postural control, which is essential to design appropriate interventions for each patient with movement disorders. SATCo-Br was considered responsive to detect changes in trunk control in the 6-month period, and consequently being able to direct strategies in the rehabilitation process.

Periodic assessments of upper limb function and trunk control can detect muscle weakness early in the disease and allow preventive interventions for contractures and minimize functional decline,^29^ as well as in monitoring the treatment of patients with DMD. A responsiveness study on theses scales in the clinical practice of health professionals is essential to indicate the frequency necessary to reassess patients with the selected measuring instrument to obtain descriptive and comparative clinical and functional information.

The limitation of the study was not being able to stratify the sample in relation to Vignos and trunk control for the analyses, not addressing the self-reported tasks of dependence and functional independence. Data were collected at a reference center for DMD that serves patients from all regions of Brazil. Despite this, the data refer to a single center.

We concluded that the JTT and PUL scales, that assess the upper limb function, and the SATCo scale, which assesses the trunk control, are responsive to detect the evolution of the disease in the 6-month interval.
